# Lignonaut: designing diverse combinatorial libraries for the exploration and annotation of lignin oligomer spaces

**DOI:** 10.1186/s13321-026-01202-9

**Published:** 2026-05-21

**Authors:** Mynta Norberg, Margareta Sandahl, Peter Spégel

**Affiliations:** Department of Chemistry, Lund University, Centre for Analysis and Synthesis, Lund, SE-221 00 Sweden

**Keywords:** Virtual, Synthesis, Feature, Library, Spectrometry, HRMS

## Abstract

Lignins are a polymeric, renewable resource with remarkable structural diversity. Research is being conducted into the valorisation of lignins into value-added products. The absence of experimental libraries, in particular for process-modified oligomers, hampers analytical feedback to these valorisation efforts. Stochastic methods for generating libraries in-silico have been proposed, but were not designed for use with popular techniques such as high-resolution mass spectrometry. To resolve this, we developed Lignonaut, which is a toolkit for designing lignin libraries through virtual combinatorial synthesis. To ensure high interpretability we also developed new, diversity-oriented nomenclature for lignin oligomers, upon which an efficient SMILES translation algorithm could be built. Libraries of up to 10^7^ oligomers could be generated, in linear time, and at a rate of 10^6^ per minute.

**Scientific contribution**

Lignonaut applies virtual combinatorial synthesis to exhaustively map lignin chemical spaces for e.g. high-resolution mass spectrometry. It is fast and can account for the high degree of isomerism in lignin oligomers, which were major limitations of previous stochastic approaches.

**Supplementary Information:**

The online version contains supplementary material available at 10.1186/s13321-026-01202-9.

## Introduction

Lignin is an aromatic biopolymer that makes up the bulk of plant dry matter alongside cellulose.[1] In pulping processes, this lignocellulose is delignified to make paper pulp, which also generates lignin-rich liquor. Due to limited demand, only 2% of such lignin side-streams are recovered as technical lignin for value-added applications,[2] such as depolymerisation into fuels and functionalised monomers and oligomers.[3] Many challenges associated with the study and use of lignins arise from their highly variable and complex compositions. Native lignins are mostly made from monomers that originate from the phenylpropanoid biosynthesis, consisting of a substituted benzene with an allyl hydroxide sidechain.[1] The three most common monomers are para-coumaryl, coniferyl, and sinapyl alcohol. Several types of linkages have been documented, which are distinguished by the moieties involved in the linkage. With the variety of monomers and linkages, branching, and inter-species variability, even native lignin is a remarkably complex biopolymer[4]. Lignin structures are also heavily altered by further processing, even by relatively mild techniques such as solvent extraction[5]. Tools for characterising the structures and compositions of lignin feedstocks and products are therefore necessary for valorisation process development, monitoring, and quality control.[3]

Many techniques have been used for characterising lignins, including gel-permeation chromatography (GPC), nuclear magnetic resonance (NMR), and high-resolution mass spectrometry (HRMS). While GPC and NMR have been used to determine the bulk distributions of molecular weights and monomer and linkage types, respectively, HRMS has been applied towards simultaneous determination of individual compounds.[3, 6] For example, by meticulously deriving fragmentation patterns from a set of model compounds, Morreel et al. (2010) could propose structures for 36 oligomers in a native lignin extract[7]. Others have taken a more comprehensive route, such as Qi et al. (2020) and Sander et al. (2023) who used Fourier-transform ion cyclotron resonance mass spectrometry (FT-ICR MS) to annotate thousands of peaks as “lignin-like” based on elemental ratios[8, 9]. Such works draw from nontargeted methodology developed within other fields of research, where tools such as van Krevelen, ring-double-bond equivalent, and Kendrick mass defect plots are common[6, 10]. Our research group previously proposed a Kendrick mass defect-based model to assign the degree of polymerisation of lignin features.[11, 12] Ultimately, the lack of experimental HRMS libraries for lignin has cemented data annotation as a major bottleneck, which is of particular concern when the true molecular composition of lignins must be known.

With beginnings tracing back to at least the 1970s[13, 14], an increasing number of studies are using computer simulation to build virtual libraries for a variety of lignins[15–17]. These efforts recently produced freely available toolkits such as Lignin-KMC[18], LigninGraphs[19], and Lignin Structure Generator[17]. However, all of these tools aim to find a small data sample (e.g. $n=100$) which optimally reproduces a set of experimentally determined bulk features. This is achieved through stochastic methods such as Markov chain Monte Carlo algorithms, which are not intended for generating the large libraries needed for HRMS data annotation. An alternative approach is diversity-oriented virtual combinatorial synthesis, which in principle is better suited for annotation as it generates exhaustive, virtual libraries as opposed to sampled datasets. Such methods have already been applied to exploring chemical spaces, and guiding the discovery and design, of drugs and polymers[20–23]. Terrell et al. (2020) demonstrated a limited, manual case of the combinatorial approach for the HRMS-based characterisation of native lignins, but eventually dismissed it as they believed it would produce an unmanageable number of oligomers[24]. Instead they heuristically expanded a small stochastic library from 100 to 1200 oligomers. As such, the development, implementation, and characterisation of diversity-oriented virtual combinatorial synthesis for lignin analysis has yet to be fully explored.

To the enable a comprehensive analysis of lignins that goes beyond classification, and to bridge the gap on the path towards experimental libraries, we developed Lignonaut. Lignonaut is a toolkit written in R, designed to be accessible, and modular enough to accommodate the rapid advances in the field. The foundation of Lignonaut is a new symbolic nomenclature encompassing non-canonical and modified monomer residues. With the support of this nomenclature, we built a diversity-oriented virtual combinatorial algorithm for synthesising unique oligomer sequences, without the need for molecular graphs. This drastically improved the performance over current stochastic approaches, allowing for exhaustive libraries (within user-defined heuristics) up to 10$^7$ oligomers to be generated in linear time and a matter of minutes, even on regular workstations. Furthermore, a dictionary-based algorithm was developed to efficiently translate the sequence names into SMILES, enabling structural validation, visualisation, and interfacing with other cheminformatic workflows.

Three proof of concept applications of Lignonaut were demonstrated: i) pre-hoc exploration of lignin chemical spaces for model development, ii) validation of predictive models and stochastic libraries, and iii) annotation of HRMS data in a targeted omics-workflow, where the process of finding tentative candidates and defining annotation confidence was greatly simplified. Lignonaut is free and open-source, and available on Codeberg for continued development.

## Computational methods

### Overview

Lignonaut was entirely written in R (version $\ge$4.5.1) because of its accessibility, and to enable seamless integration with already existing cheminformatics and mass spectrometry workflows. Dependencies were limited to the data.table (version 1.17.6), stringi (1.8.7) and Rfast (2.1.5.1) packages at the time of writing. Microbenchmark (1.5.0) was used for measuring compute times. The packages ggplot2 (3.5.2), ggside (0.3.1), FactoMineR (2.11) and factoextra (1.0.7) were used for data visualisation.

Most functions in Lignonaut are collected in a composite function, which acts as a wrapper that allows the user to conveniently select between a large number of plausible monomeric residues and linkages, and then iterate up to a chosen degree of polymerisation. Lignonaut is not distributed as a package, to simplify development through enabling users to add new monomeric residues and linkages by simply altering a set of .csv files. A set of quality control functions are also included, which help ensure that any new additions are working as intended.

### Symbolic notation for diverse oligomers

As is the case with many biological copolymers (such as DNA and peptides), symbolic notation is practically necessary for communicating sequences. The currently used one-letter symbol notation is incomplete, as it is limited to the three or four most common monomers. For Lignonaut, we needed to specify non-canonical and modified monomers and linkages. Much like for amino acids, a multi-symbol notation is necessary for making room for uncommon monomers, and to maintain legibility. The first step was therefore to design a nomenclature to support this.

**Fig. 1:**
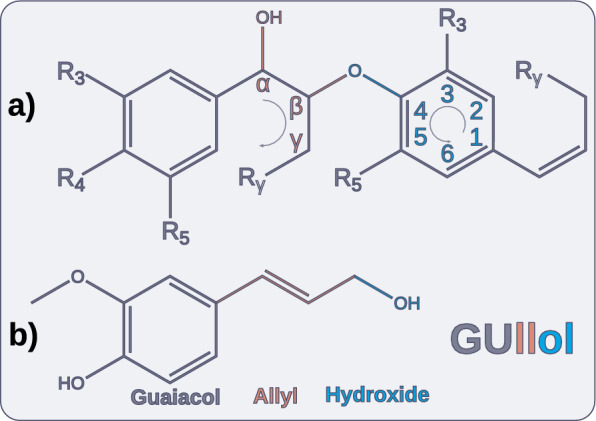
**a** The typical alphanumeric vertices used for lignin, here illustrated across a $\upbeta$-O-4 linked lignin dimer. **b** Mnemonic derivation of the descriptive, six-letter symbol for coniferyl alcohol residue, using the three characteristic moieties of phenylpropanoids

Conventionally, single-letter symbols are used for lignin monomers, and an alphanumeric vertex-to-vertex notation for linkages that specifies which atoms that connect the monomers (illustrated in Fig. 1a). We noted that the scope of this sequence notation is inherently limited, because it specifies the monomers that supposedly formed the sequence, as opposed to the monomer residues actually found in it. While this might be sufficient for many use-cases, we believe that a symbolic notation must be descriptive as opposed to retrospective to capture the diverse sequences found in both native and process-modified lignins. To enable this, we opted for a system using six-letter symbols for monomer residues (not monomers). The conventional linkage notation however could be used as is.

This descriptive notation is based on a nomenclature that annotates the three characteristic moieties of canonical, phenylpropanoid residues: substituted benzene, side-chain, and side-chain substituent (illustrated in 1b). Using the conventional linkage notation, we can then denote e.g. the vanillin dimer (divanillin) as GUmeal[5-5]GUmeal, where the vanillin residue symbol (GUmeal) was derived from the semi-arbitrary mnemonic "guaiacol methylene aldehyde". Systematically denoting such compounds has not been possible before. Several hundred other residues were similarly named using this nomenclature, and tabulated for use with Lignonaut. For non-phenylpropanoid monomer residues (such as tricin), an arbitrary notation of i.e. four capital letters could be used. Further details on this nomenclature will be available with the Lignonaut documentation.

### Virtual synthesis of diverse combinatorial libraries

To enable the construction of combinatorial lignin libraries, a database of plausible monomer residues was constructed. At the time of publication, 400 residues were included in this database. It contains identifiers including the six-letter symbols, alongside descriptors such as atom counts, taxonomical classifiers, and reactivity variables. A sample of this database is available in Supplementary Table A1.

**Fig. 2:**
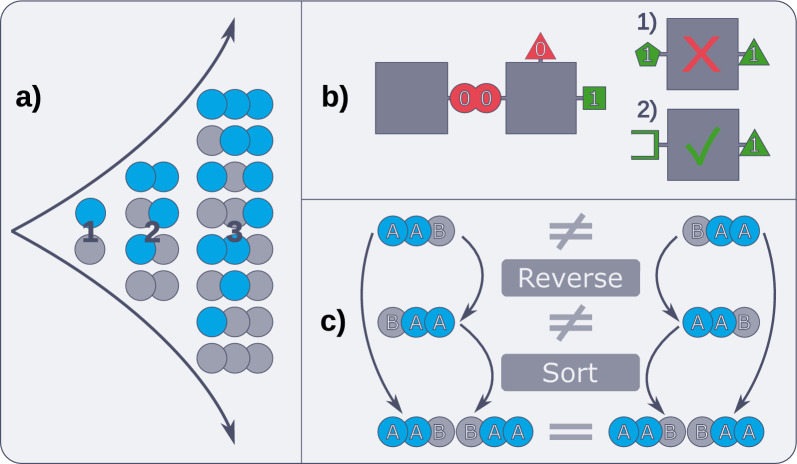
Illustration of the key steps in the synthetic algorithm. **a** Visualisation of the combinatorial algorithm up to degree 3. For clarity, only two monomers (blue and gray) and one linkage were used here. **b** Boolean variables (illustrated by the colored shapes) are used to keep track of significant reactive moieties, allowing for the rest of the structure to be treated as a “black box”. Here, the dimer endgroup can only successfully combine with monomer 2, and monomer 1 is rejected from the vector. **c** The sorted vector of a sequence and its reverse are combined to form a convergent identifier for duplicate removal

Lignonaut uses linking functions to perform all operations associated with the formation of a particular linkage. The linking functions take two inputs, one table of monomer residues, and one table of sequences with degree *n* (the *n*-mers, often starting from $n=1$) that will be extended by those monomer residues. Boolean variables are used to track whether certain moieties are available for reaction or not. These variables are specified for each monomer residue, and are then updated for each iteration depending on the effect of the linking function (see Fig. 2b). For example, to form a $\upbeta$-O-4 linkage, the endgroup of the *n*-mer must have a vinyl group, and the monomer residue a 4-OH. Any inputs that do not fulfill these criteria are filtered out, ensuring that only constitutionally viable oligomers are formed.

After filtration, the linking function applies a combinatorial algorithm to find all possible combinations (as illustrated in Fig. 2a). The *n*-mer table is replicated as a table by the number of rows in the monomer table. Conversely, the monomer table is replicated row-wise by the number of rows in the *n*-mer table. This yields two tables with the same number of rows, whose columns can be operated on as vectors to yield an exhaustive table of the (*n*+1)-mers. As the monomer residues form the new endgroups, the linking function uses their boolean variables as the basis for the (*n*+1)-mers, adjusted for moieties consumed during the reaction. The atom counts of the tables are also added, including correction terms to account for any atoms gained or lost during the reaction. A full list of all 12 linkages available in Lignonaut at the time of publication, along with their filtration criteria and correction terms are found in Supplementary Table B2.

If the linkage is asymmetrical with respect to reversal, all of these steps are repeated for the reverse of the linkage. While this produces a large number of mirror duplicates, such pairs will not produce the same sequence in the next iteration. To illustrate, the sequence AAB is the mirror duplicate of BAA, but after C is added in the next iteration, then AABC and BAAC are no longer duplicates. As such, mirror duplicates at iteration *i* are identified and removed only after iteration $i+1$ is finished. To achieve this, a set of operations that produces the same identifier for both duplicates is needed. In Lignonaut this is achieved by forming a vector of both the forward and reverse sequences (including linkage direction), and then sorting them alphabetically (see Fig. 2c). Because both duplicates will converge on the same identifier, exactly one of the duplicates can then be discarded.

### Dictionary-based algorithm for SMILES translation

**Fig. 3:**
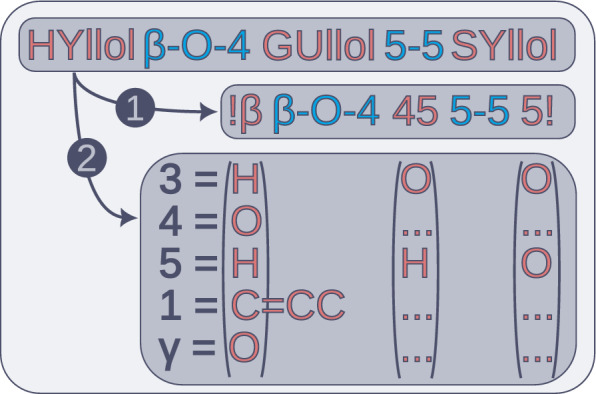
Illustration of how a valid oligomer sequence can be decomposed into (1) a backbone with generic residues, which only describes connectivity, and (2) substituent information. The alphanumerals represent positions, and the triple dots signify row-wise repeated entries

While cheminformatics tools for processing SMILES strings are available, such as RDKit and pysmiles, they rely on molecular graphs as inputs. To reduce costs and avoid external dependencies altogether, we developed an algorithm for translating our sequence names directly into SMILES. The algorithm exploits that the vertices in the linkage notation not only specifies the intermonomer connectivity, but also the intramonomer connectivity. This means that any given sequence can be decomposed into a backbone and substituents, similar to peptides (see Fig. 3). In the backbone sequence, the monomer residues are replaced with generalised backbone residues derived from the adjacent linkage edges. As such, they only contain connectivity information. The low number of such residues made the use of manually maintained static dictionaries for SMILES translation feasible. Using these, the backbone can then be translated into SMILES snippets which contain placeholder substrings, which are then substituted with substituent and ring index information. A full description of the algorithm is provided in Supplementary Figure C3.

## Experimental section

An Indulin AT lignin, purchased from MeadWestvaco (Charleston Heights, SC, USA), was dissolved in 90:10 (v/v) tetrahydrofurane:water to 10 mg/mL, of which 2.0 $\upmu$L was injected on an Agilent Technologies 1260 Infinity supercritical fluid chromatography (SFC) system. The SFC was in turn coupled to a timsTOF Pro 2 (Bruker Daltonics) with electrospray ionisation (ESI) operated in positive mode. The SFC was equipped with a Torus 1-AA column (130Å, 1.7 $\upmu$m, 2.1 mm by 100 mm, Waters) heated to $40^\circ$C, the flow rate was 1.25 mL/min, and the backpressure regulator set to 130 bar. Carbon dioxide (99.9993% purity, Linde Gas) was used with methanol (VWR, LCMS grade) as cosolvent (B), starting at 2% B until 2 min, then 12% B by 3.5 min, 25% B by 7 min, 35% B by 9.5 min, and back down to 2% B by 10 min. The makeup was 5% water in methanol with 10 mM ammonia, at a flow rate of 0.85 mL/min. ESI-L Low Concentration Tuning Mix (Agilent Technologies) was used for both the mobility calibration, and mass calibration, using the high precision calibration option (HPC in timsControl). The SFC was controlled with OpenLAB CDS ChemStation, and the HRMS data with timsControl. The acquisition method was based on the 4D-Lipidomics Application method (Bruker Daltonics), with a scan range of m/z 150–1500, mobility range of 0.55−1.90 Vs/cm$^2$, and parallell-accumulation serial-fragmentation (PASEF) enabled. The nebuliser pressure of 3.0 bar, dry gas flow and temperature of 10.0 L/min and $250^\circ$C respectively, and a Focus Pre TOF Transfer Time of 90 $\upmu$s, were altered from the defaults. Feature identification was performed in TASQ with default settings (version 2022.1.1 9353, Bruker Daltonics). Further experimental details are provided in Section 4.3.

## Results and discussion

### Benchmarking of algorithms

With the number of algorithms in Lignonaut, each having qualitatively different inputs, determining a single computational complexity is not possible. LigninGraphs and Lignin-KMC faced a similar issue, and chose to report the time complexity for the growth of a single polymer[18, 19]. They determined this empirically by measuring runtimes across different input sizes. While the case of single polymer growth is not relevant to diversity-oriented methods, a similar empirical approach could be applied to Lignonaut.

The computational complexities of all algorithms were determined, e.g. for the linking, duplicate removal, and SMILES translation. Full details of the experimental designs and results are available in Supplementary Section D. In summary, all algorithms ran in linear time (*O*(*n*)) at moderate input sizes ($n>1000$). However, the apparent time complexity of the SMILES translation algorithm was higher in more realistic use-cases, where e.g. input size and degree of polymerisation grow together. All of the features of Lignonaut (linking, duplicate removal, and SMILES translation) are combined in a composite function, for convenience and optimisation purposes. When using this nested function, the SMILES translation step, and in particular the ring-indexing step, was the slowest. Despite the room for improvement, the composite function was able to output at a rate of approximately $10^6$ unique oligomers per minute. This suggested that Lignonauts combinatorial approach was on the order of $10^4$ times faster than the state-of-the-art stochastic approaches (for generating diverse libraries)[18, 19].

Given that the output of each linkage iteration grows by $2n^2$, memory costs in Lignonaut were inevitably much more important to limit. While it is possible to allocate virtual memory in R, out-of-memory operations are inevitably slow. A typical Lignonaut library will contain symbolic name sequences, atom counts, exact masses, and SMILES strings. A data row or oligomer from such a library will roughly use 300 bytes. With 32 GB random-access memory and a constant space complexity, the maximum number of oligomers with SMILES that can be handled within memory would be 100 million. However, because of necessary duplicate generation and the need to copy data for some operations, the practical limit in terms of unique oligomers is closer to 50 million. Although it is not possible to preselect a certain output length, we have successfully been able to build libraries with tens of millions of oligomers. This can be compared to the LGS dataset (n = 60 000) by Eswaran et al. (2022), which at the time of writing was the largest published lignin dataset.[17] As will be demonstrated in later sections, this is sufficient for various applications. However, there is an inevitable trade-off between high structural diversity and high degrees of polymerisation. As such, we left it up to the user to select monomeric residues and linkages based upon the features of interest, reasonable assumptions about the sample, or other heuristic constraints. This also allows the user to design and explore chemical spaces relevant for their particular application.

### Leveraging lignin chemical spaces for model and library validation

**Fig. 4:**
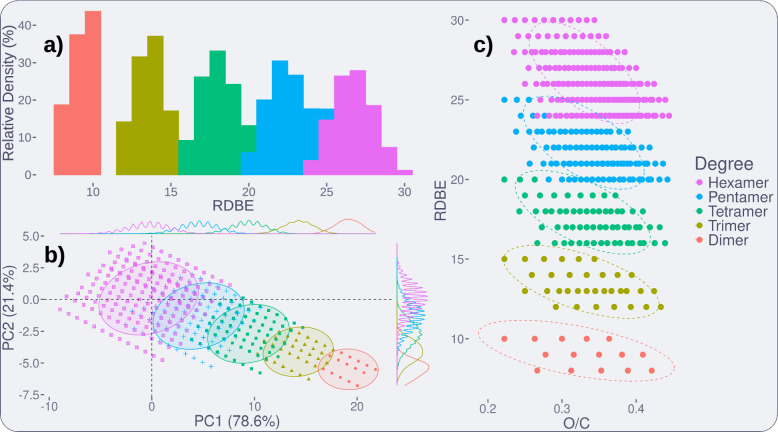
All of the following figures were generated from the same dimer to hexamer Lignonaut library, as described in this section. **a** RDBE values of dimers (red) to hexamers (magenta), as relative densities (100% within groups). A bin width of 1 was used, as only integer RDBE values were found. The bars are stacked and not overlayed. **b** PCA plot of six KMD definitions, with the first and second principal components, and 95% confidence ellipses. Overlap is already evident between the dimers and trimers. **c** Plot of RDBE and O/C atom ratio, with 95% confidence ellipses. The first overlap is between pentamers and hexamers

A useful property of exhaustive libraries is that they form chemical spaces with well-defined boundaries. As such, they can be used in the development and validation of classification models. For example, Tammekivi et al. (2024) recently proposed a simple ring-double bond equivalent (RDBE) model for predicting the degree of polymerisation of oligomers[25]. This was based on the inference that the RDBE values for monomers to tetramers should typically fall in the ranges of 4–7, 8–11, 12–15, and 16–19, respectively. To test this, a library stopping at hexamers was built with Lignonaut, using H, G, and S monomers, and $\upbeta$-O-4, $\upalpha$-O-4, $\upbeta$-$\upbeta$, $\upbeta$-1, 5-5, $\upbeta$-5, and 5-O-4 linkages. This selection is highly conventional and simple, and as such served as an effective sanity check. In the returned library (n = $4\cdot 10^6$), the determined RDBE ranges for dimers to hexamers were 8–10, 12–15, 16–20, 20–25, and 24–30, respectively (see Fig. 4a). While this adds validity to RDBE-based classification up to tetramers, a trend of a growing overlap beyond this is evident. The first point of overlap is at 20 RDBEs, where tetramers with two $\upbeta$-$\upbeta$-linkages (7%) may be confused with pentamers. This overlap is bound to start earlier if residues with even higher RDBE values, such as cinnamic acid-like residues or tricin, are added. This overlap was demonstrated with experimental data in previous work from our group.[11]

We also applied this kind of treatment to study the Kendrick mass defect (KMD)-based classification model for degree of polymerisation, which was previously described in our group by Prothmann et al.[11, 12, 26] The six KMD definitions, based on common lignin fragments (CO, CHO, CH_2_O, CH_3_O, CO_2_, CHO_2_, and C_5_H_5_O), were calculated for the same library used for the RDBE case, and used in principal component analysis (PCA, see Fig. 4b). Similar to the RDBE case, the overlap rapidly increases with degree of polymerisation, but here the overlap is already seen between dimers and trimers. To our surprise, simply combining RDBE in a plot with the O/C atom count ratio (from Van Krevelen diagrams) produced 95% confidence ellipses with the first overlap occurring between pentamers and hexamers (Fig. 4c). This combination is sensible, as RDBE correlates to degree of polymerisation and linkage types[25], and the O/C atom counts ratio correlates to types of monomer residue (e.g. their degree of oxidation or functionalisation). We believe such plots to be more interpretable, reproducible, and useful for general data visualisation tasks. This could also be applied for simple prediction models based on spanned areas of the confidence ellipses, for any library and confidence level of choice.

**Fig. 5:**
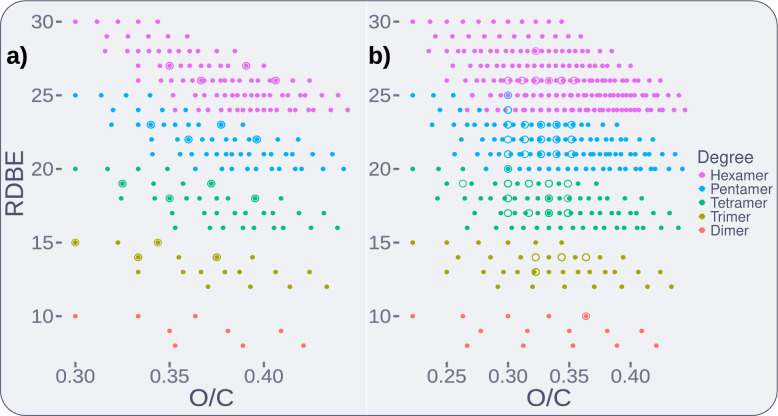
**a** Subset of the LGS dataset up to hexamers ($n = 244$) from Eswaran et al. (2020) in circles, alongside a G and S dataset built with Lignonaut ($n = 2\cdot 10^5$) in points. **b** Subset of a combination of the LigninGraphs Pine, Poplar, and Miscanthus demo-datasets up to hexamers ($n = 223$) in circles. A highly conventional H, G, and S library built with Lignonaut ($n = 4 \cdot 10^6$, in points) still manages to span a larger chemical space

Moving on, we applied this plot towards visualising the differences between combinatorial and stochastic chemical spaces. For this we found two publicly available datasets, the LGS dataset published by Eswaran et al. (2020), and a set of five demo-datasets from https://ligningraphs.readthedocs.io (both downloaded on March 19, 2024)[17, 19]. For comparison with the LGS dataset we generated a similar dataset in Lignonaut, using G and S monomers, and the previously mentioned seven conventional linkages. For the actual LGS dataset, we excluded heptamers and above ($n = 244$) to keep the size of the Lignonaut library manageable ($n = 2\cdot 10^5$). These are plotted together in Fig. 5a, from which it is evident that the LGS dataset does not contain any dimers, and only covers four points per degree of polymerisation for the chosen range. While the LGS dataset reaches up to 25-mers, this comparison shows that the chemical diversity within each degree of polymerisation is highly limited. To form a more diverse stochastic dataset, we combined the five LigninGraphs demo-datasets (Pine branched and unbranched, Poplar branched and unbranched, and Miscanthus unbranched) into one. Once again, heptamers and above were excluded, leaving a small subset ($n = 223$). For this comparison, a Lignonaut dataset ($n = 4 \cdot 10^6$) which also included H monomers was used, e.g. the same “conventional dataset” from the RDBE and PCA cases described previously. In this case (see Fig. 5b), several non-overlapping points could be identified. These are due to the absence of reduced linkage variants and branched oligomers in the Lignonaut dataset. Branching was not yet implemented in Lignonaut at the time of writing, and while the reduced linkage variants were, the picture was clear in any case. Despite having simpler inputs, the chemical space spanned by the Lignonaut library was much larger. Explaining why the stochastic algorithms produce such limited diversity was outside the scope of this study. However, from this comparison we crucially were able to demonstrate that the combinatorial approach was well suited towards producing the chemically diverse datasets needed for both modelling and annotation purposes. We therefore moved on to investigating how Lignonaut can be used in data annotation.

### Combinatorial libraries for annotation of lignin HRMS data

Many classification workflows, such as the RDBE and KMD-based models explored in the previous section, alongside atomic ratio and van Krevelen plots, were developed for the annotation of high-resolution mass spectrometry (HRMS) data. [8, 9, 12, 25] We applied Lignonaut in a targeted omics-like workflow for the annotation of Indulin AT lignin data (see Supplementary Figures E4 and E5). A selection of characteristic Indulin AT features were used with Lignonaut to generate a library ($n=8.3 \cdot 10^6$, dimers to hexamers). These included distinct features such as $\upbeta$-sulfanyl substituents and $\updelta$-valerolactone linkages[27], while also prioritising features that are more prevalent[28]. In total, sixteen monomer residues and seven linkages were used, which are found in Supplementary Figure E6. From this library, a suspect list with just 4735 unique masses could be derived. The suspect list was used with Bruker TASQ, excluding features with poor isotopic fit (mSigma > 200 ppm), and $\Updelta$m/z values outside ±3. This left 404 features, or 172 unique masses with experimental isomer counts between 1–19 (resolved in the ion-mobility stage), and library match isomer counts (tentative candidates) of 1–2011. 70% of isomers had counts below 20. No pentamers or hexamers were detected.

This library matching is comparable to and improves upon the “lignin-likeness” classifier of Qi et al. (2020) and Sander et al. (2023), [8, 9], since the matches are based on plausible chemical structures, instead of typical atomic ratios. With respect to the annotation confidence levels by Schymanski et al. (2014), where lower levels indicates higher confidence, this would be a level 4 annotation.[29] However, Lignonaut also enables the annotation of tentative structures (e.g. confidence level 3) by matching the feature masses with library masses. Terrell et al. (2020) similarly used a virtual library as the basis for assigning tentative candidates to 80 features, but deliberately limited these to two structures per feature, as they were not able to identify the number of isomers with their stochastic approach[24]. Prothmann et al. (2020) were also unable to propose a number of tentative candidates, and instead relied on classification results and partial structural matches for confidence level 3[12]. Given the high isomerism of lignin oligomers, such structural assignments come with a high unknown uncertainty. Schymanski et al. (2014) described annotation confidence level 3 as a “grey zone”, with confidence sublevels that should be determined on a per-study basis[29]. As demonstrated by the previous examples, no consensus for how to determine such sublevels exists for lignin oligomers. In our Indulin AT library, both the mean and standard deviation of the number of isomers grew by an order of magnitude with each degree of polymerisation. They reached 3486 and 195%, respectively, for hexamers. We have consistently seen similar figures with other Lignonaut libraries, suggesting that isomerism is important to take into account.

**Fig. 6:**
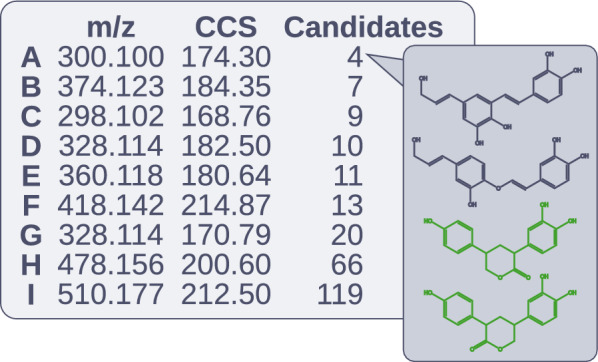
A set of features was sampled based on peak intensity, shape, and MS$^2$ data. The number of candidates was highly variable, and feature A was therefore prioritised. The MS$^2$ data (see Figure E4) was well aligned with the previously unreported dimers in green

To demonstrate how knowledge of isomerism can be used to guide structure elucidation, we sampled seven features which had principal ions with large peak areas and gaussian peak shapes, low peak interference, and rich MS$^2$ data. The number of constitutional isomers (e.g. tentative candidates) for these features ranged between 4 and 119, and were mostly previously unreported dimers and trimers (see Fig. 6). Given our proof-of-concept scope, we chose a feature with four tentative structures for further analysis. Supplementary Table E4 shows a summary of the MS$^2$ results. Common neutral losses such as [M+H-15] were not detected, consistent with the lack of methoxy groups in all candidates. While the [M+H-18] loss might suggest alkanol, this is also seen with the alkoxy group of five-membered lactones, alongside other detected losses such as [M+H-HCOOH][30]. We were also able to propose other plausible fragments consistent with the $\updelta$-valerolactone linked candidates. However, further experiments making use of i.e. MS$^n$ are necessary for a complete annotation confidence level 2, especially to distinguish between the regioisomeric $\updelta$-valerolactone links. While such experiments are outside of the scope of this article, having these candidates to guide design the MS$^n$ experiments makes it a promising next step. Other promising applications not touched upon in this study include using the generated SMILES strings with already established analytical and cheminformatic pipelines, such as fingerprint prediction with SIRIUS, as demonstrated by Tammekivi et al. (2024).[25] Altogether, we believe that this approach has the potential of speeding up the population of much needed experimental lignin libraries, especially when supported by the other cheminformatic tools included with Lignonaut.

## Conclusions

A toolkit was developed which included a monomer residue database, nomenclature for diverse lignin oligomers, and virtual combinatorial library generation supported by linkage functions, duplicate removal, SMILES translation, and validation functions. All without any major dependencies. Being the first demonstration of a purely combinatorial approach for virtual lignin synthesis, it was characterised and compared with established stochastic approaches. The combinatorial approach was $10^4$ times faster in terms of number of oligomers per minute, and scaled well due to a linear time complexity. The exponential, in-memory growth of generated libraries led to a practical upper limit of around 50 million oligomers on personal computers. This was $10^3$ times larger than the largest lignin library to date. The use of heuristics enabled the design of libraries with a limited size, without necessarily losing features of interest.

Proof of concept applications were explored, including the visualisation and characterisation of lignin chemical spaces, and the structural annotation of HRMS data. Lignonaut-generated libraries were used to assess two models for degree of polymerisation, including a KMD-based model previously proposed by our own group. While both models showed a rapidly growing overlap, the RDBE-based model had excellent resolution up to tetramers. Stochastically generated datasets from LigninGraphs, and the LGS dataset, were compared to equivalent Lignonaut libraries. The combinatorial libraries generated by Lignonaut spanned a much larger chemical space than all of the stochastic datasets, suggesting that Lignonaut was well suited for experimental applications such as de-novo data annotation for HRMS.

A targeted omics-like workflow was explored, where a Lignonaut library designed for the chosen lignin was used as the basis for a suspect list. Each exact mass was associated with plausible candidate structures, providing more information than previous approaches based on atomic ratios. This not only provided a starting point for structural elucidation, but also crucial information on the highly variable degree of isomerism. It also provided a concrete definition of confidence sublevels for tentative structure annotations for comprehensive lignin analysis workflows. Future aspects included using combinatorial libraries for the design of MS$^n$ experiments, and integration of SMILES into previously established analytical and cheminformatic workflows.

## Supplementary Information

Additional file 1.

## Data Availability

Lignonaut is available on Codeberg under an MIT license, at https://codeberg.org/myntanorberg/Lignonaut, including all testing scripts. Libraries described in the work are not made available, as they can be reproduced per instructions given in the Supplementary Information

## Abbreviations

*FT-ICR MS*: Fourier-transform ion cyclotron resonance mass spectrometry
*GPC*: Gel permeation chromatography
*GUllol*: Coniferyl alcohol residue (“guaiacyl allyl alcohol”)
*GUmeal*: Vanillin residue (“guaiacyl methylene aldehyde”)
*HRMS*: High-resolution mass spectrometry
*HYllol*: Paracoumaryl alcohol residue (“4-hydroxybenzyl allyl alcohol”)
*KMD*: Kendrick mass defect
*NMR*: Nuclear magnetic resonance
*PASEF*: Parallell Accumulation Serial Fragmentation
*PCA*: Principal component analysis
*RDBE*: Ring-double bond equivalent
*SFC*: Supercritical-fluid chromatography
*SMILES*: Simplified Molecular Input Line Entry System
*SYllol*: Sinapyl alcohol residue (“syringyl allyl alcohol”)
