# Supplementary material for: Lignonaut: designing diverse combinatorial libraries for the exploration and annotation of lignin oligomer spaces
Source: J Cheminform. 2026 May 21;18:68. doi: 10.1186/s13321-026-01202-9 (PMC13200465; doi:10.1186/s13321-026-01202-9)
Supplement: Supplementary file 1 — Additional file 1. [file 13321_2026_1202_MOESM1_ESM.pdf]

Supplementary information.

Appendix A Lignin monomer residues

Appendix B Lignin reactivity

Special terminology was introduced for more intricate linkages to avoid confusion, such as the "open-open" variant of the beta-beta linkages (βO-Oβ), and valerolactone linkage (1-vl-1). Other linkages such as the stilbene linkages (1-C=C-1 and 5-C=C-1) and enol-ether linkage (1-C=C-O-4) fully describe the edges and the atoms and bonds between them, similar to 4-O-5 or 5-5. We pronounce i.e. "1-C=C-1" as "one C C one", similar to how other linkages are pronounced. No criteria was used for the 1-vl-1 linkage at the time of publication due to its obscurity. It was instead generated arbitrarily by removing the sidechains and forming the link from the 1-positions. The approaches for the linkage functions including how the correction terms are calculated might change over time. See the Codeberg repository for further and current details.

Appendix C SMILES algorithm

Appendix D Computational complexity

To study the computational complexity of the linkage functions, the entry for coniferyl alcohol (which possessed all types of reactivity at the time of writing) was replicated to form input tables of different lengths, for which the runtime for all seven basic linkage functions was measured (β-O-4, α-O-4, β-β, β-1, β-5, 5-5 and 4-O-5). The order of the linkage functions and input sizes was randomised, and each was respectively measured in triplicate. This gave 810 times in total, to which power regression models ($an^b = t$ ms) were fitted. From this it was determined that the time complexities for

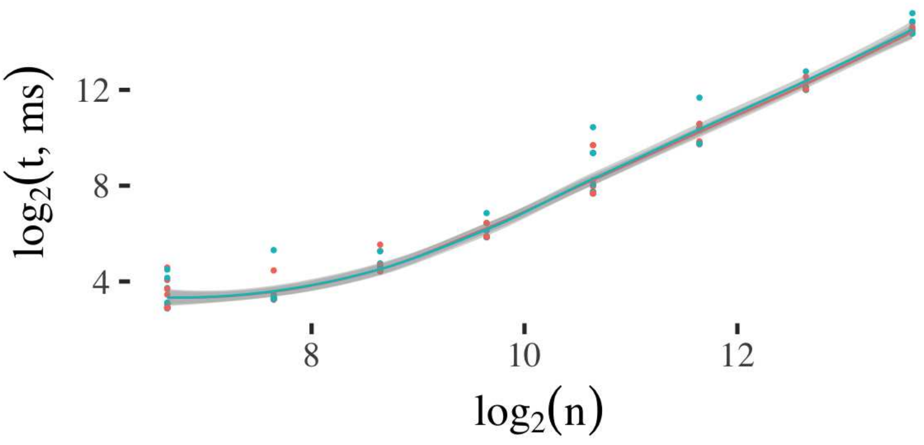

Fig. D1 Time complexity experiments for the asymmetric β-O-4 (red) and α-O-4 (blue) linkage functions, which suggest $O(n)$ at high input sizes. The overlap between the two fitted curves suggests a high repeatability.

the linkage functions follow three distinct patterns, with asymmetrical linkages (i.e. β-O-4) suggesting the highest time complexity of $O(N^{1.8})$. However, from Figure D1 it is evident that the non-linearity is only evident with $n < 1000$, and that the algorithm reaches an oblique asymptote and runs in linear time ($O(n)$) for $n > 1000$, which could be considered a moderate input size.

D.1 Complexity with respect to degree

The complexity with respect to degree was not noteworthy. E.g. the runtime for link.bo4(100-mer, 1-mer) was 8.8 ms, compared to the 4.6 ms of link.bo4(1-mer, 1-mer).

D.2 Time complexity of duplicate removal algorithm

The duplicate removal algorithm was $O(n)$ in time for dimers and trimers, with a small increase between them. This suggests that the time complexity may increase slightly with degree of polymerisation.

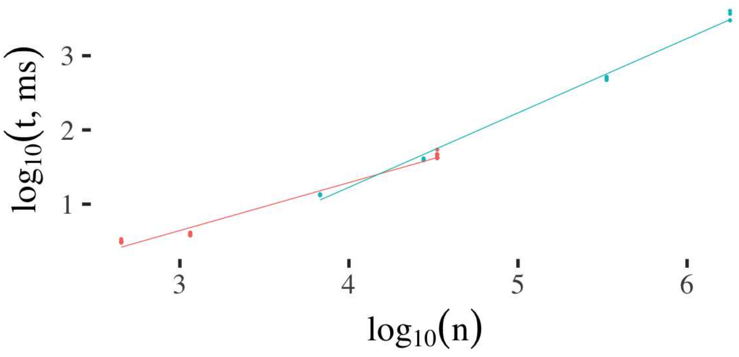

Fig. D2 Time complexity experiments for the duplicate removal algorithm, applied to dimers (red) and trimers (blue). Both suggest $O(n)$ across several orders of magnitude.

D.3 Time complexity of SMILES algorithm

The time complexity of the SMILES algorithm was studied with respect to both input size, and degree of polymerisation. For the input size experiments, a table of approximately 1000 tetramers was generated using H, G, S and β-O-4 linkages, repeated to create 6 longer tables up to approximately 100'000. The translation time was measured in triplicate at each point. The power regression model gave an exponent of 1.008, which indicated a slight non-linear increase over several orders of magnitude (see Figure D3a), but in practice this gives a time complexity of $O(n)$. However, as seen in Figure D3b, the rate of translation also decreases with degree of polymerisation. Combined, the complexity is likely higher, approaching either $O(2n)$ or $O(n^2)$.

Appendix E High-resolution mass spectrometry

Appendix F Guide to using Lignonaut

Software can undergo frequent changes, especially when young. An up to date guide for using Lignonaut will always be available at https://codeberg.org/myntanorberg/Lignonaut (hereafter referred to as "the Codeberg").

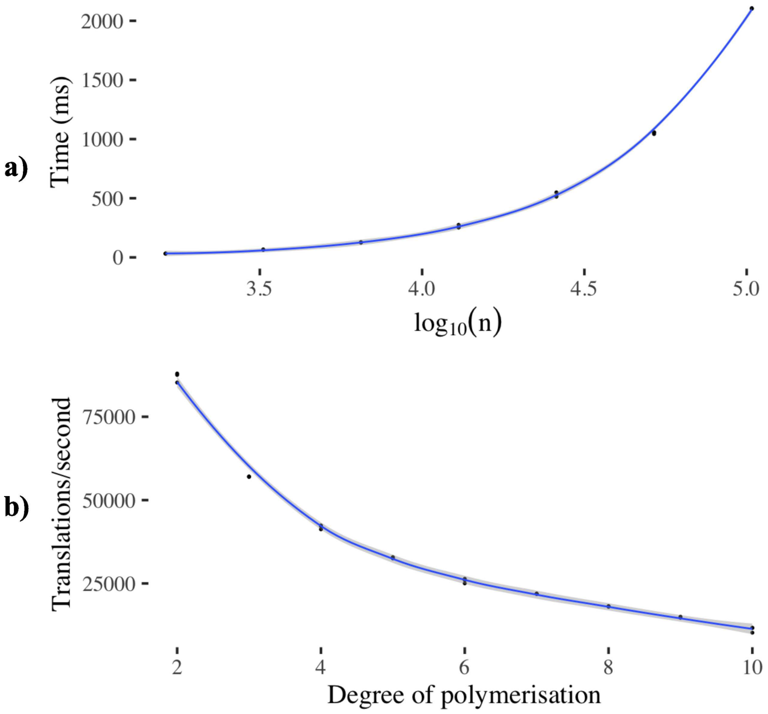

Fig. D3 (a) Time complexity of the SMILES algorithm with respect to input size (n) is $O(n^{1.008})$ or approximately $O(n)$, but (b) The translation rate also decreases with degree of polymerisation, and when combined produces an apparent non-linear time complexity.

At the time of publication, Lignonaut is not distributed as a regular R package. Instead it is uploaded to Codeberg, which is a host for code repositories (similar to GitHub). To install Lignonaut, the repository simply needs to be downloaded from the Codeberg. A guide for how to do this is found in the README.md file, which is also visible as a description box on the Codeberg.

Below are instructions for how to reproduce the libraries used in the article. These examples showcase how to apply **lignonaut.build_oligomers()**, which is a wrapper function for all key functions in Lignonaut. The arguments to this function are a set of monomers and linkages. The examples showcase two prebuilt monomer sets: an H, G and S set, and the indulin set. It also shows how to build custom sets, and how to select linkages.

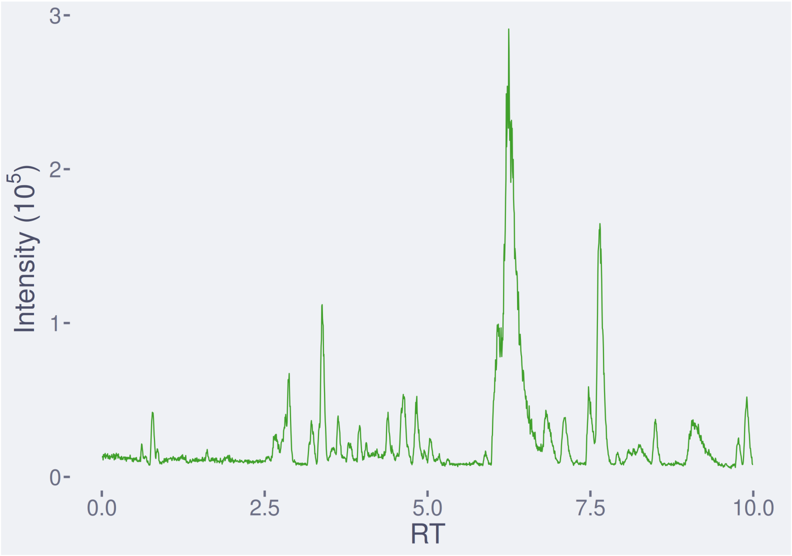

Fig. E4 Base-peak chromatogram of Indulin AT lignin.

Number of recursions (recs) also needs to be specified. Combining monomers with monomers forms dimers, and is performed within one recursion. In the next recursion, dimers will be combined with monomers to form trimers, and so on. As such, setting **recs=2** means that the function would finish at trimers. All of the examples also set **SMILES=FALSE**, which disables SMILES translation to save some time and memory. This is useful (and often necessary) when building very large libraries.

Further examples will be availabe on the Codeberg.

F.1 H, G, and S with seven linkages, up to hexamers

Here we can make use of the **lignonaut.monomers_hgs** subset for convenience. Because of size, library-wide SMILES translation is turned off to avoid surpassing RAM limits. But individual ones can still be translated with the **lignonaut.name_to_smiles()**.

Try this with **recs=4** or even **recs=3** before proceeding to **recs=5**, especially if you only have 16 GB of RAM or less.

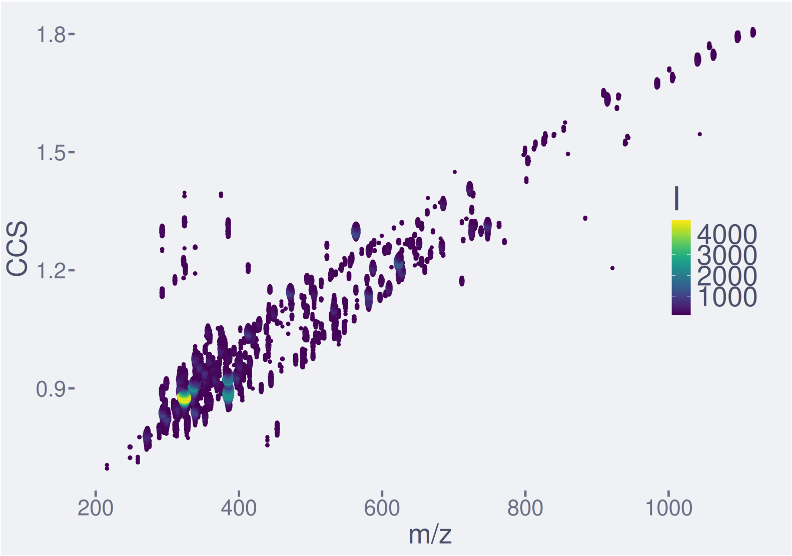

Fig. E5 Heatmap of Indulin AT lignin, with points with intensities (I) below 100 removed. The point size was also set to increase with I, to further improve the legibility.

```
hgs <- lignonaut.build_oligomers(
  lignonaut.monomers_hgs,
  recs=5,
  links=c("lignonaut.link_bo4",
          "lignonaut.link_ao4",
          "lignonaut.link_b1",
          "lignonaut.link_55",
          "lignonaut.link_b5",
          "lignonaut.link_4o5",
          "lignonaut.link_bb"),
  SMILES=FALSE)
```

F.2 G and S variant

This subset can be created as follows, and then replace **lignonaut.monomers_hgs** above.

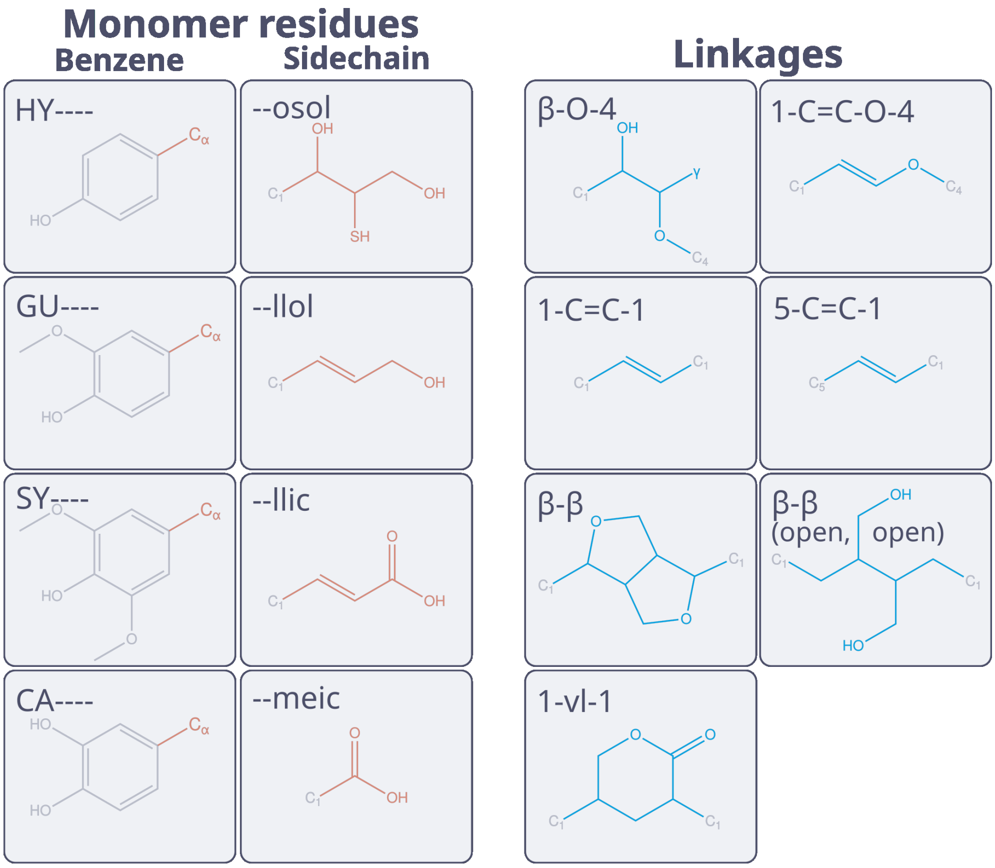

Fig. E6 Summary of the structural moieties used to generate the Indulin AT library used for HRMS annotation, where C_i refers to the carbon that the moiety connects to. A total of sixteen monomer residues from Lignonaut were used, here reduced to four substituted benzenes and sidechains for the sake of visualisation.

```
lignonaut.monomers_gs <- lignonaut.monomers_raw[
  short == "GUllol" | short == "SYllol"
  ][,
    -c("ring", "sidechain", "name", "short",
    "smiles", "rc", "rh", "ro", "rs")
  ]
```

F.3 Indulin library

This is based on the already available subset called **lignonaut.monomers_indulin**, which is created in **initialise_lignonaut_workspace.R**. Different linkages were used here, but otherwise the call is similar to the previous ones.

```
ind <- lignonaut.build_oligomers(
  lignonaut.monomers_indulin,
  recs=5,
  links=c("lignonaut.link_bo4",
          "lignonaut.link_bb",
          "lignonaut.link_1cc1",
          "lignonaut.link_5cc1",
          "lignonaut.link_1vl1",
          "lignonaut.link_1cco4",
          "lignonaut.link_bOOb"),
  SMILES=FALSE)
```

Table A1 Small sample of the Lignonaut monomer table, showcasing all sidechains and some columns for the monomer residues with a phenol ring. The reactive moieties described in Supplementary Section B is also included. The full and most recent table is available in the Codeberg repository.

ring	sidechain	short	c	h	o	s	has.vinyl	has.5H	has.4OH	has.gOH
phenol	hydrogen	HY	6	6	1	0	FALSE	TRUE	TRUE	FALSE
phenol	hydroxyl	HYol	6	6	2	0	FALSE	TRUE	TRUE	FALSE
phenol	methyl	HYme	7	8	1	0	FALSE	TRUE	TRUE	FALSE
phenol	benzylic alcohol	HYmeol	7	8	2	0	FALSE	TRUE	TRUE	FALSE
phenol	benzylic aldehyde	HYmeal	7	6	2	0	FALSE	TRUE	TRUE	FALSE
phenol	benzylic ketone	HYmeon	8	8	2	0	FALSE	TRUE	TRUE	FALSE
phenol	benzylic acid	HYmeic	7	6	3	0	FALSE	TRUE	TRUE	FALSE
phenol	ethyl	HYet	8	10	1	0	FALSE	TRUE	TRUE	FALSE
phenol	vinyl	HYvi	8	8	1	0	TRUE	TRUE	TRUE	FALSE
phenol	propyl	HYpr	9	12	1	0	FALSE	TRUE	TRUE	FALSE
phenol	propylic alcohol	HYprol	9	12	2	0	FALSE	TRUE	TRUE	TRUE
phenol	propylic methyl ether	HYprmx	10	14	2	0	FALSE	TRUE	TRUE	FALSE
phenol	propylic ethyl ether	HYprex	11	16	2	0	FALSE	TRUE	TRUE	FALSE
phenol	mercapto-propanol alcohol	HYosol	9	12	3	1	FALSE	TRUE	TRUE	TRUE
phenol	propene	HYpe	9	10	1	0	FALSE	TRUE	TRUE	FALSE
phenol	propenyl	HYll	9	10	1	0	TRUE	TRUE	TRUE	FALSE
phenol	allylic alcohol	HYllol	9	10	2	0	TRUE	TRUE	TRUE	TRUE
phenol	allylic sulfonyl hydrox-ide	HYllsa	9	10	4	1	TRUE	TRUE	TRUE	FALSE
phenol	allylic aldehyde	HYllal	9	8	2	0	TRUE	TRUE	TRUE	FALSE
phenol	allylic ketone	HYllon	10	10	2	0	TRUE	TRUE	TRUE	FALSE
phenol	allylic acid	HYllic	9	8	3	0	TRUE	TRUE	TRUE	FALSE
phenol	allylic methyl ester	HYllms	10	10	3	0	TRUE	TRUE	TRUE	FALSE
phenol	allylic ethyl ester	HYlles	11	12	3	0	TRUE	TRUE	TRUE	FALSE

Table B2 Summary of the 12 linkages that are currently available in Lignonaut, along with their associated criteria, and correction terms used in calculating the new sum formula. The criteria are separated into to groups, associated with either monomer residue 1 or 2, or an oligomer endgroup and monomer residue. R represents the partial sum of the side chain, e.g. the residue linked to the 1 position, that is lost in the formation of the linkage. R refers to either group 1 or 2, whereas R_n refers to group n only.

Linkage	Criteria group 1	Criteria group 2	Correction term(s)
β-O-4	α=β	4-OH	+O
α-O-4	α=β	4-OH	+O
β-β	α=β & γ-OH	α=β & γ-OH	-H_2
βO-Oβ	α=β & γ-OH	α=β & γ-OH	-H_2
β-5	α=β	4-OH & 5-H	-H_2
β-1	α=β	α=β	+OH - R
5-5	5-H	5-H	-H_2
4-O-5	4-OH	5-H	-H_2
1-C=C-1	α=β	α=β	+C_2H_2 - 2 x R
5-C=C-1	5-H	α=β	-(R_2 + C_2H)
1-C=C-O-4	α=β	4-OH	-(R_1 + C_2H_3) - H_2
1-vl-1	NA	NA	+$C_5H_6O_2$ - 2 x R

Table C3 Step-by-step summary of how the SMILES translation algorithm in Lignonaut would translate the dimer HYllol[α-O-4]GUllol. Some other types of linkages require extra steps, which are not described here.

Operation	Output
Split sequence into residual units	HYllol<br>α-O-4<br>GUllol
Find generic residues (backbone)	!α<br>α-O-4<br>4!
Translate to generic SMILES (backbone)	ci(cc(*3*)c(*4*)c(*5*)ci)<br>O<br>cic(*5*)cc(cci*3*)*1*
Fill in 1-, 3-, 4-, and 5-substituents	ci(cc()c(O)c()ci)C(C(O)CO)<br>O<br>cic()cc(cciOC)/C=C/C*γ*
Fill in γ-substituents	ci(cc()c(O)c()ci)C(C(O)CO)<br>O<br>cic()cc(cciOC)/C=C/CO
Enumerate rings	c1(cc()c(O)c()c1)C(C(O)CO)<br>O<br>c2c()cc(cc2OC)/C=C/CO
Clean up	c1(ccc(O)cc1)C(C(O)CO)<br>O<br>c2ccc(cc2OC)/C=C/CO
Concatenate	c1(ccc(O)cc1)C(C(O)CO)Oc2ccc(cc2OC)/C=C/CO

Table E4 Summary of MS^2 data for analyte with neutral mass 300.10 ($C_{17}H_{16}O_5$), RT of 5.32, and a CCS of 174.3. The ion structures are given in SMILES, and can be parsed with i.e. Chemdraw. The support is tentative and merely provided as a proof of concept for a Lignonaut-enabled de-novo annotation workflow.

m/z	ΔmDa	*I*	Loss	Ion	Support
301.		<LOD	[M+1]		
286.		<LOD	CH3/CH4		Not expected
283.1006	-3.6	50	H2O	Oc1ccc(cc1)C(C)C=C(C#[O+])c2cc(O)c(cc2)O	Crotti (2004), catechol
273.		<LOD	CO		Catechol, minor
271.		<LOD	CH2O	Oc1ccc(cc1)C(C)C=C(C#[O+])C2C=CC(C=C2)=O	Not expected
265.1220	-3.6	30	2 H2O		Crotti (2004), catechol
257.		<LOD	CO2		Not expected
255.2120	-110.0	40	HCOOH	Oc1ccc(cc1)C(C)C=[C+]c2cc(O)c(cc2)O	Crotti (2004), catechol
252.8532	-146.3	30	48		C2H6O1?
245.1177	-36.3	60	56		C3H4O1?
241.0894	-2.9	190	C2H4O2	[OH+]=C1C=CC(C=C1)CC=cc2cc(O)c(cc2)O	Thermodynamic
236.0450		30	65		
226.0612		50	75		
217.0841		50	84		
213.0899	1.7	310	C3H4O3	[OH+]=C1C=CC(C=C1)CC=CC2=CC=C(O)C2	Thermodynamic
209.0584	23.0	160	92		C6H4O1?
197.0602		60	104		
181.0673	-17.2	300	Vinylphenol	C=[O+]C(O)=Cc1ccc(c(O)c1)O	Keto-enol to retro-Diels Alder.
170.0717		40	131		
167.0815		20	134		
164.0614		60	137		
161.0648		70	140		
153.0698		80	148		
